# Contralateral vs. Ipsilateral Approach to Superior Hypophyseal Artery Aneurysms: An Anatomical Study and Morphometric Analysis

**DOI:** 10.3389/fsurg.2022.915310

**Published:** 2022-05-25

**Authors:** Balkan Sahin, Serdar Onur Aydin, Mehmet Ozgur Yilmaz, Tahsin Saygi, Sahin Hanalioglu, Goktug Akyoldas, Oguz Baran, Talat Kiris

**Affiliations:** ^1^ Microsurgical Neuroanatomy Laboratory, Koc University Hospital, Istanbul, Turkey; ^2^ Department of Neurosurgery, Faculty of Medicine, Hacettepe University, Ankara, Turkey; ^3^ Department of Neurosurgery, Koc University Hospital, Istanbul, Turkey; ^4^ Department of Neurosurgery, American Hospital, Istanbul, Turkey

**Keywords:** superior hypophyseal artery, aneurysm, contralateral, ipsilateral, approach, microsurgical anatomy

## Abstract

**Introduction:**

Surgical clipping of superior hypophyseal artery (SHA) aneurysms is a challenging task for neurosurgeons due to their close anatomical relationships. The development of endovascular techniques and the difficulty in surgery have led to a decrease in the number of surgical procedures and thus the experience of neurosurgeons in this region. In this study, we aimed to reveal the microsurgical anatomy of the ipsilateral and contralateral approaches to SHA aneurysms and define their limitations via morphometric analyses of radiological anatomy, three-dimensional (3D) modeling, and surgical illustrations.

**Method:**

Five fixed and injected cadaver heads underwent dissections. In order to make morphometric measurements, 75 cranial MRI scans were reviewed. Cranial scans were rendered with a module and used to produce 3D models of different anatomical structures. In addition, a medical illustration was drawn that shows different sizes of aneurysms and surgical clipping approaches.

**Results:**

For the contralateral approach, pterional craniotomy and sylvian dissection were performed. The contralateral SHA was reached from the prechiasmatic area. The dissected SHA was approached with an aneurysm clip, and maneuverability was evaluated. For the ipsilateral approach, pterional craniotomy and sylvian dissection were performed. The ipsilateral SHA was reached by mobilizing the left optic nerve with left optic nerve unroofing and left anterior clinoidectomy. MRI measurements showed that the area of the prechiasm was 90.4 ± 36.6 mm^2^ (prefixed: 46.9 ± 10.4 mm^2^, normofixed: 84.8 ± 15.7 mm^2^, postfixed: 137.2 ± 19.5 mm^2^, *p* < 0.001), the distance between the anterior aspect of the optic chiasm and the limbus sphenoidale was 10.0 ± 3.5 mm (prefixed: 5.7 ± 0.8 mm, normofixed: 9.6 ± 1.6 mm, postfixed:14.4 ± 1.6 mm, *p* < 0.001), and optic nerves’ interneural angle was 65.2° ± 10.0° (prefixed: 77.1° ± 7.3, normofixed: 63.6° ± 7.7°, postfixed: 57.7° ± 5.7°, *p*: 0.010).

**Conclusion:**

Anatomic dissections along with 3D virtual model simulations and illustrations demonstrated that the contralateral approach would potentially allow for proximal control and neck control/clipping in smaller SHA aneurysm with relatively minimal retraction of the contralateral optic nerve in the setting of pre- or normofixed chiasm, and ipsilateral approach requires anterior clinodectomy and optic unroofing with considerable optic nerve mobilization to control proximal ICA and clip the aneurysm neck effectively.

## Introduction

The superior hypophyseal artery (SHA) arises from the posteromedial surface of the internal carotid artery just distal to the distal dural ring. This terminology was first used by Day (1, 2). The SHA is responsible for the arterial supply of the pituitary stalk, optic nerves, and optic chiasm (2).

SHA aneurysms, together with carotid cave, posterior carotid wall, and carotid–ophthalmic aneurysms, are referred to as paraclinoid aneurysms (3, 4). Although SHA aneurysms are rare, they cause subarachnoid hemorrhage due to their intradural localization. Although the surgical treatment of SHA aneurysms has decreased with the development of endovascular treatment techniques, it still seems difficult to treat patients with low dome-to-neck ratios endovascularly.

First, the contralateral approach to bilateral carotid–ophtalmic aneurysms was defined by Yaşargil, and then, case series in the literature were shared by many authors (5–11). The surgical approach and clipping of SHA aneurysms are challenging for many reasons such as close proximity to important neurovascular structures, narrow surgical corridor, and difficulty in proximal control. Literature is scarce regarding the conditions and limits of the ipsilateral and contralateral approach for SHA aneurysms.

In this study, we aimed to reveal the microsurgical anatomy of the ipsilateral and contralateral approaches to SHA aneurysms and define their limitations *via* morphometric analyses of radiological anatomy, three-dimensional (3D) modeling, and surgical illustrations.

## Materials and Methods

### Preparation of Specimens

In this study, five silicone-injected cadaver heads were used. Cadavers were fixed with a 10% formalin solution for at least 3 weeks. Silicone injection in all whole-head specimens was performed using the technique described by Shimizu et al. (12). Dissections were performed under ×6–40 magnification using a Zeiss Surgical Microscope (Carl Zeiss AG, Oberkochen, Germany). During the study period, all specimens were kept in a 75% alcohol solution. Three-dimensional images of each step of the dissection were obtained.

### Radiological Examinations

In order to make morphometric measurements (perchiasmatic area, interneural angle, interneural length, etc.) in prefixed, normofixed, and postfixed chiasm types, we performed a thorough retrospective search on the institutional PACS for scans of patients 18–65 years who underwent any magnetic resonance imaging (MRI) of the head. We then reviewed patient charts and applied the following exclusion criteria: history of any brain or orbit tumor, enucleation of eye, optic nerve lesions, enlargement or atrophy of the optic nerve, raised intracranial pressure (treated or not), and ischemia, hemorrhage, or atrophy along the optic pathway. We included patients who had indications for MRI other than optic nerve lesions or any cranial mass effect lesions. Scans with poor visualization of the optic nerve were excluded.

A total of 75 MRIs with 18 prefixed, 36 normofixed, and 21 postfixed chiasms that met the eligibility criteria were selected. Retrospectively evaluated studies included MRI images from a 1.5 T MRI system (Magnetom Siemens Altea, Germany). Imaging parameters included FOV180, slice thickness of 0.8, TR-5.83, TE-2.53, frequency of 63.68 MHz, NEX-1, bandwidth of 399 Hz/Px, and measurements were done using a 64-bit medical image viewer for OS X. Horos DICOM viewer, an open-source software based upon OsiriX, was used to view the sectional MRI images and perform morphometric measurements.

**Figure 1:**
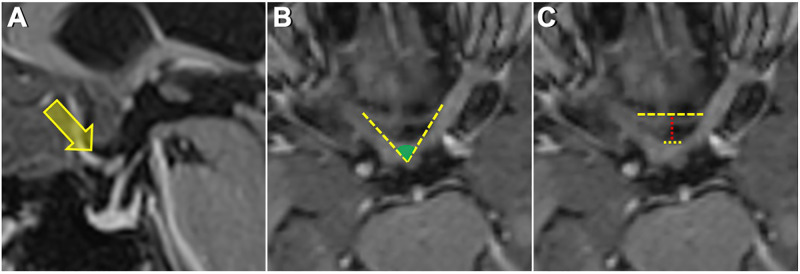
(**A**) Sagittal MRI image showing a prefixed chiasm—overlying tuberculum sella. (**B**) Measurement of the interneural angle on axial reformatted images along the intracranial optic nerves. (**C**) Upper dashed yellow line shows the distance between the bilateral optic nerves at the entrance to the optic canal. The lower dashed yellow line shows the distance between the optic nerves just before they form the optic chiasm. The dashed red line shows the distance from the anterior aspect of the optic chiasm to the limbus sphenoidale.

**Figure 2:**
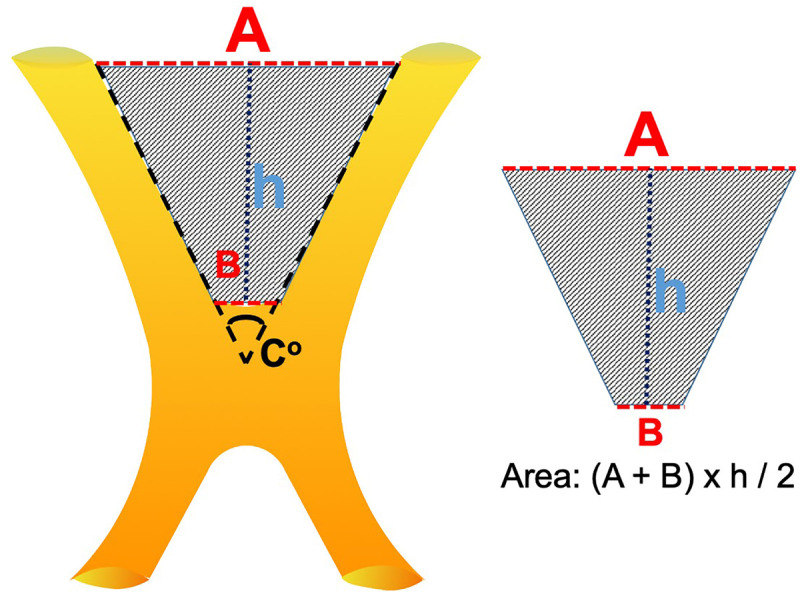
Graphic representation of the prechiasmatic space. *h*, the distance from the anterior aspect of the optic chiasm to the limbus sphenoidale; A, the distance between the bilateral optic nerves at the entrance to the optic canal; B, the distance between the optic nerves just before they form the optic chiasm. Prechiasmatic area (scanned area) calculated by using the trapezoid area formula: [(*A* + *B*) × *h*]/2.

In sagittal images evaluated for the location of the optic chiasm, the optic chiasm overlying the tuberculum sellae was termed as prefixed, the optic chiasm overlying the diaphragm sellae was termed as normal, and the optic chiasm overlying the dorsum sellae was termed as postfixed chiasm (Figure 1A). The angle between the medial (inner) border of the intracranial portion of bilateral optic nerves, termed the interneural angle, and between the medial aspect of the optic tracts, termed the optic tract angle, were calculated from the axial reformatted images along the intracranial optic nerves and optic tracts (Figure 1B). Parameters *h*, *A*, and *B* were defined to estimate the size of the prechiasmatic cistern. Parameter *h* (mm) is the distance between the anterior margin of the optic chiasm and the limbus sphenoidale, parameter *A* (mm) is the distance between the bilateral optic nerves at the entrance to the optic canal, and parameter *B* (mm) is the distance between the bilateral optic nerves forming the optic chiasm. Parameters *h*, *A*, and *B* were measured on an MRI slice made parallel to the long axis of the optic nerves (Figure 1C). The estimated size of the prechiasmatic area was calculated using the following formula: (*A* + *B*) × *h*/2 (Figure 2).

### Reconstruction of the Virtual 3D Simulation Model

All 3D planning and modeling studies were carried out with Mimics Innovation Suite 22.0 software (Materialise, Leuven, Belgium). Briefly, DICOM files of MRI or computed tomography (CT) scans were imported into Mimics. Radiological images were visualized on axial, coronal, and sagittal planes. The masking process was undertaken using hounsfield unit (HU) values on two-dimensional radiological images. Segmentation of various structures was done according to anatomical borders. Different imaging sequences were used for the segmentation of different intracranial structures: CT for bone, time of flight (TOF) MR angiography for arteries, and T2-weighted MRI for the optic nerve. All CT and MRI scans were merged and aligned with the Align Global Registration module. Surface rendering was used to produce 3D models of different anatomical structures. Then, a design module (3-matic 14.0, Materialise, Leuven, Belgium) was used for fine-tuning and detailed modeling. For this specific study, we artificially created variations to the existing anatomy (pre- and postfixed optic nerve, addition of SHA, addition of aneurysms of different sizes). This allowed us to simulate a surgical scenario by freely rotating, positioning, and trimming the model virtually. The optic nerve was made transparent to visualize the underlying SHA.

### Statistical Analysis

The Statistical Package for the Social Sciences for Windows (version 15.0; IBM Corporation, Armonk, NY, USA) was used for statistical analysis. Results were expressed as mean, standard deviation, and percentage scores wherever appropriate. Measurements were compared in the prefixed, normofixed, and postfixed chiasms using a one-way ANOVA test. A *p*-value <0.05 was used to denote statistical significance.

## Results

### Anatomical Aspects of the Ipsilateral and Contralateral Approach

#### Contralateral Approach

**Figure 3:**
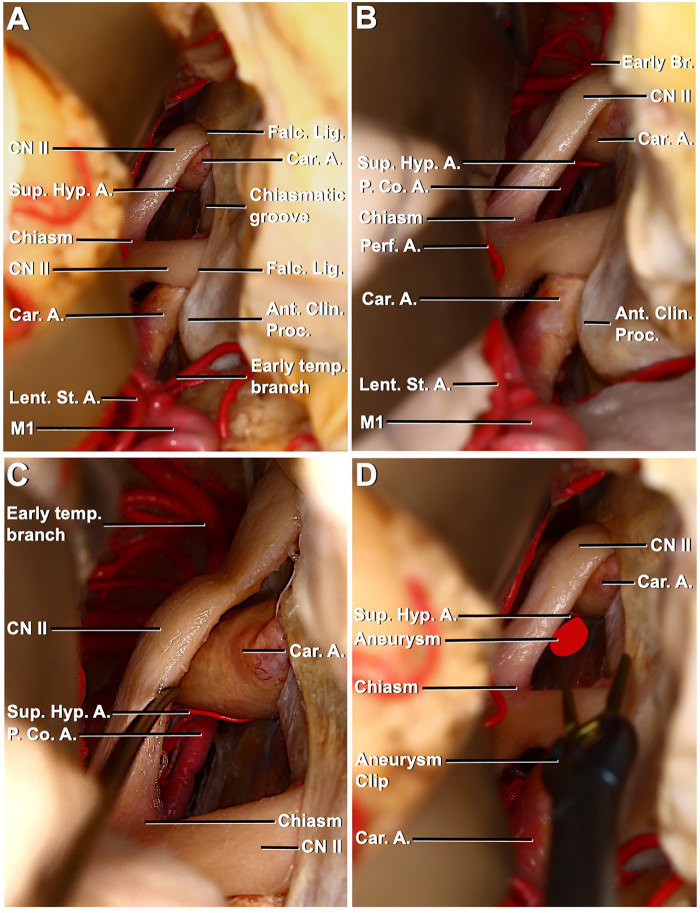
Right pterional exposure of the preoptic chiasm and circle of Willis, contralateral approach to the left superior hypophyseal artery. (**A**) Right frontotemporal bone flap is elevated, and the dura is opened. The right frontal and temporal lobes have been retracted to expose the right carotid artery entering the dura medial to the anterior clinoid process. Right–left optic nerves, optic chiasma, prechiasmatic area, left carotid artery, and left superior hypophyseal artery are visible. (**B**) Exposure has been extended between the chiasm and frontal lobe to the left posterior communicating artery (PCoA) left superior hypophyseal artery arising from the ophthalmic segment. (**C**) PCoA, the course of the left superior hypophyseal artery, is exposed through left optic nerve mobilization. (**D**) Aneurysm of the left superior hypophyseal has been illustrated and approached with an aneurysm clip to mimic the contralateral surgical approach.

The right pterional approach was used in the first stage of dissection. After dural opening, proximal sylvian dissection was performed. MCA bifurcation, M1, lenticulostriate arteries, early temporal branch, ipsilateral and contralateral ICA, carotid bifurcation, A1, anterior clinoid process, ipsilateral and contralateral optic nerves, prechiasmatic area, and optic chiasma were identified (Figures 3A,B). The SHA originating from the medial surface of the ophthalmic segment of the ICA was identified by a slight elevation of the left optic nerve with the opening of the prechiasmatic cistern. Again, in the same exposure, the left PComA was in the field of view. It was observed that the SHA extended toward the pituitary gland, pituitary stalk, and optic nerve. Terminal branches from the artery were seen to extend from below the chiasm to the floor of the third ventricle (Figures 3C,D).

#### Ipsilateral Approach

**Figure 4:**
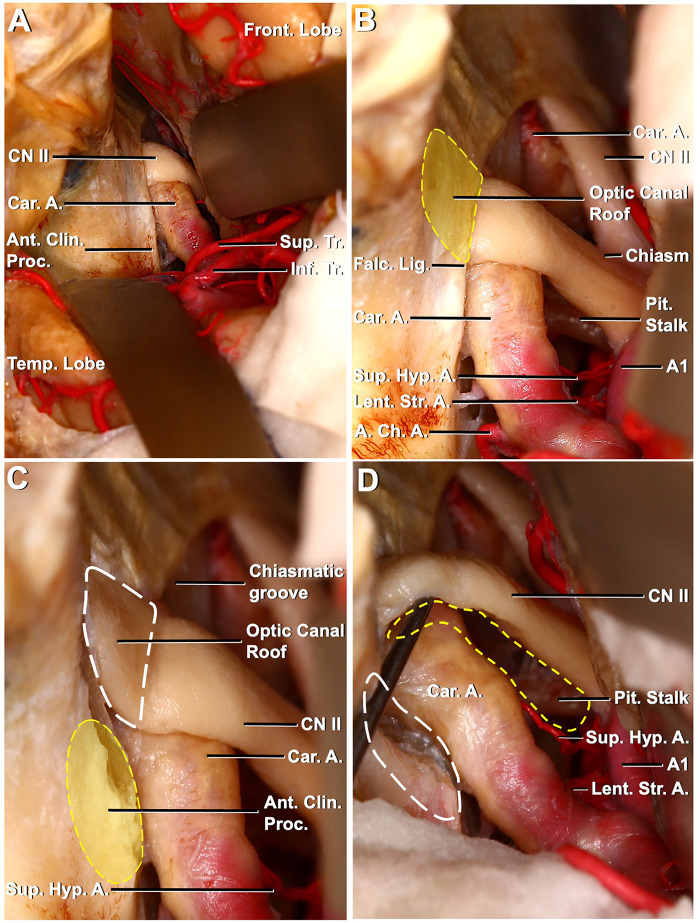
Left pterional exposure of the preoptic chiasm and circle of Willis, the ipsilateral approach to the left superior hypophyseal artery. (**A**) Left frontotemporal bone flap is elevated, and the dura is opened. The left frontal and temporal lobes have been retracted to expose the left carotid artery entering the dura medial to the anterior clinoid process. The left optic nerve, anterior clinoid process, and prechiasmatic area are seen. (**B**) Exposure has been extended between the chiasm and the frontal lobe. Pituitary stalk and left superior hypophyseal artery are seen between the left optic nerve and left carotid artery. The optic canal roof is depicted by a dashed yellow line. (**C**) Optic canal roof, depicted by a dashed white line, is removed. The anterior clinoid process is depicted by a dashed yellow line. (**D**) Anterior clinoid process, depicted by a dashed white line, is removed and the left optic nerve is mobilized. The corridor for the ipsilateral approach to the left superior hypophyseal artery is seen (dashed yellow line) between the left optic nerve and the mobilized left carotid artery.

In the left pterional approach to a different specimen, following sylvian dissection, MCA bifurcation, superior and inferior trunk, anterior clinoid process, carotid artery, and optic nerve were revealed (Figure 4A). When the dissection was extended, the SHA was identified in the left opticocarotid triangle. Subsequently, intradural anterior clinoidectomy was performed. The falciform ligament is defined and incised. Cutting the falciform ligament was beneficial for mobilizing the optic nerve. The optical canal is defined, and the optic roof is removed (Figures 4B,C). After optic unroofing, the optic nerve was mobilized. After mobilization, it was observed that the SHA originated from the medial of the ICA-ophthalmic segment, extended medially and posteriorly, and a surgical corridor was created (Figure 4D).

**Figure 5:**
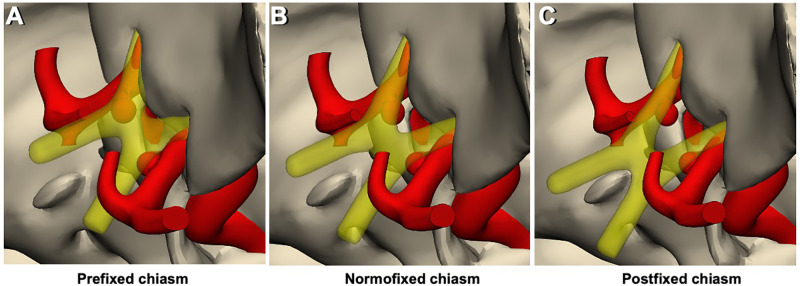
3D models of chiasm types and the relationship of the prechiasmatic area – aneurysms. (**A**) Prefixed chiasm type, regardless of its size and shape, the aneurysm remains below the optic chiasm. Consequently, there is not enough space for the contralateral approach. (**B**) Normofixed chiasm type provides sufficient space for the contralateral approach, depending on the shape and size of the aneurysm. (**C**) Postfixed chiasm type provides sufficient space for the contralateral approach, more than the normofixed chiasm type, depending on the shape and size of the aneurysm.

**Figure 6:**
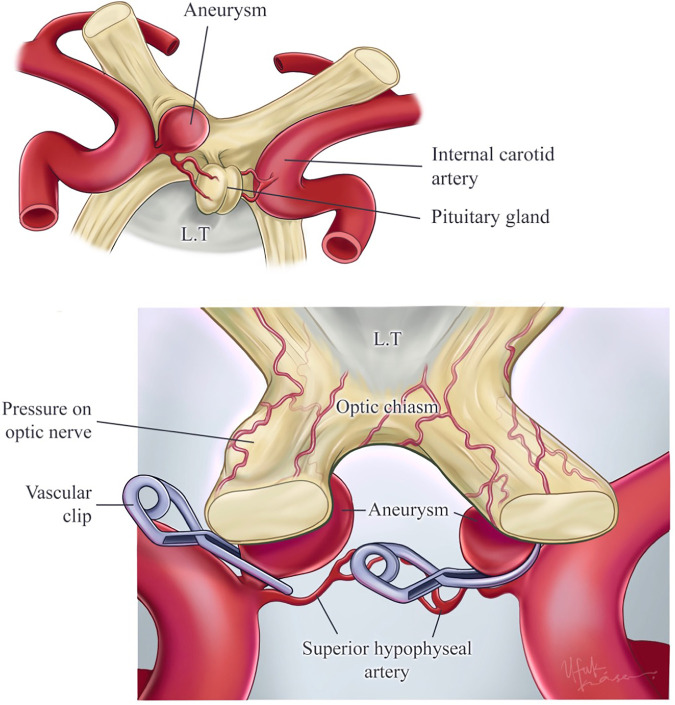
Illustration of superior hypophyseal artery aneurysms, contralateral–ipsilateral aneurysm clipping. The right superior hypophyseal artery has aneurysms that are not suitable for the contralateral approach because of the aneurysm size and the challenge of reaching the aneurysm dome. Also, this aneurysm’s pressure on the optic nerve has been illustrated. This aneurysm is suitable for the ipsilateral approach. On the other hand, the illustrated left superior hypophyseal artery aneurysm is suitable for the contralateral approach because of the aneurysm size (small). Also, this aneurysm allows reaching the aneurysm dome by the contralateral approach. Printed with permission from Ufuk Köse.

Figures 5, 6 show the 3D model and artistic depiction of the ipsilateral and contralateral approaches. Virtual 3D model simulations included bilateral SHA aneurysm scenarios and represented three different chiasm variations based on real measurements (mean values obtained in the following section were used for simulations).

Anatomic dissections along with 3D virtual model simulations and illustrations demonstrated that (i) the contralateral approach would potentially allow for proximal control and neck control/clipping in smaller SHA aneurysm with relatively minimal retraction of the contralateral optic nerve in the setting of pre- or normofixed chiasm and (ii) the ipsilateral approach requires anterior clinodectomy and optic unroofing with considerable optic nerve mobilization to effectively control proximal ICA and clip the aneurysm neck.

### Morphometric Analysis

A total of 75 patients [37 males (49.3%) and 38 females (50.7%)] were included in the study. Chiasm types were 18 (24%) prefixed, 36 (48%) normofixed, and 21 (28%) postfixed. Prefixed chiasm group’s age was 41.8 ± 10.6 years, normofixed chiasm group’s age was 37.9 ± 13.9 years, and postfixed chiasm group’s age was 38.9 ± 12 years. There was no significant difference in sex and age distribution groups.

The distance between the bilateral optic nerves at the entrance to the optic canal in all patients was 14.7 ± 1.9 mm, in the prefixed group was 12.8 ± 1.6 mm, in the normofixed group was 14.8 ± 1.5 mm, and in the postfixed group was 16 ± 1.4 mm, and there was no significant difference (Figures 1, 2).

The distance between bilateral optic nerves where they form the optic chiasm in all patients was 13.1 ± 0.9 mm, in the prefixed group was 3.5 ± 0.9 mm, in the normofixed group was 2.9 ± 0.8 mm, and in the postfixed group was 3.1 ± 1.0 mm, and there was no significant difference (Figures 1, 2).

The distance between the anterior aspect of the optic chiasm to the limbus sphenoidale in all patients was 10.0 ± 3.5 mm, in the prefixed group was 5.7 ± 0.8 mm, in the normofixed group was 9.6 ± 1.6 mm, and in the postfixed group was14.4 ± 1.6 mm, and there was no significant difference between distribution groups (Figures 1, 2) (*p* < 0.001).

**Figure 7:**
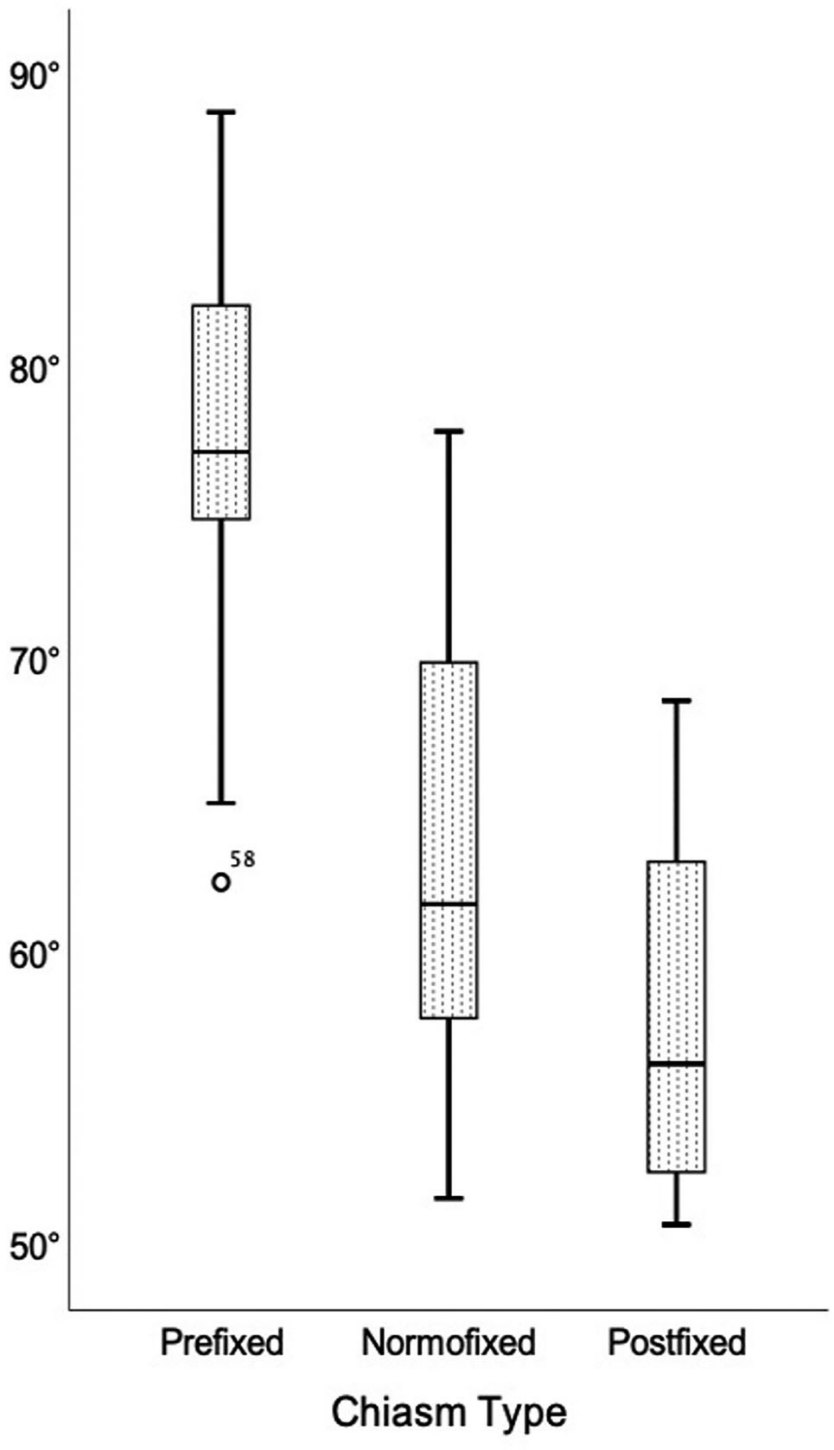
Comparison of the interneural angle in prefixed, normofixed, and postfixed optic chiasm types.

The optic nerves' interneural angle in all patients was 65.2° ± 10.0°, in the prefixed group was 77.1° ± 7.3°, in the normofixed group was 63.6 ° ± 7.7°, in the postfixed group was 57.7° ± 5.7°, and there was no significant difference between distribution groups (Figure 7) (*p* = 0.010).

The length of the medial side of the right optic nerve in all patients was 11.3 ± 3.0 mm, in the prefixed group was 8.0 ± 1.1 mm, in the normofixed group was 10.8 ± 1.5 mm, and in the postfixed group was 15.1 ± 1.5 mm, and there was no significant difference between distribution groups (*p < *0.001).

The length of the medial side of the left optic nerve in all patients was 11.2 ± 2.8 mm, in the prefixed group was 7.9 ± 1.0 mm, in the normofixed group was 10.9 ± 1.6 mm, and in the postfixed group was 14.7 ± 1.2 mm, and there was no significant difference between distribution groups (*p < *0.001).

**Figure 8:**
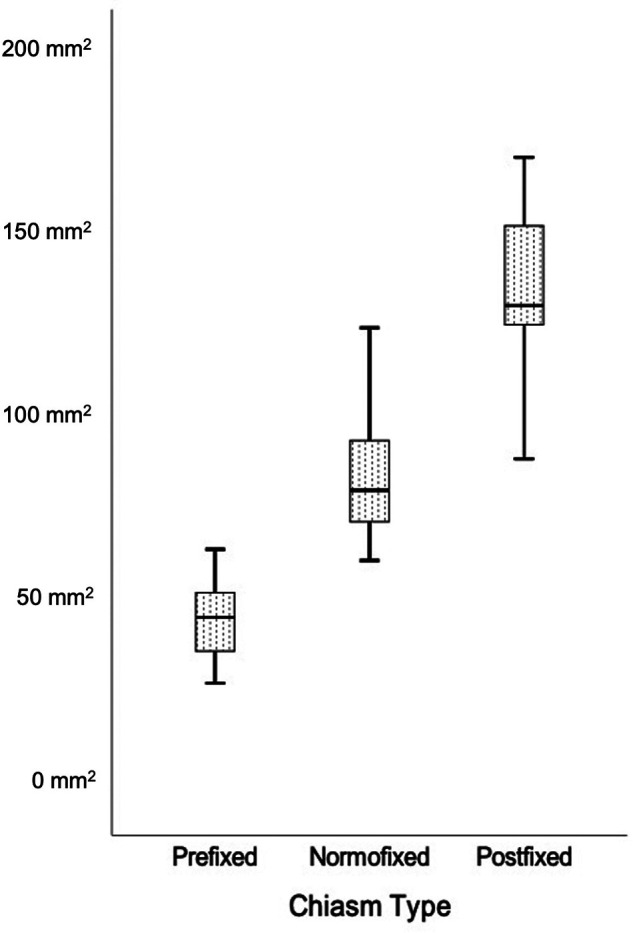
Comparison of the prechiasmatic space in prefixed, normofixed, and postfixed optic chiasm types.

The area of the prechiasm in all patients was 90.4 ± 36.6 mm^2^, in the prefixed group was 46.9 ± 10.4 mm^2^, in the normofixed group was 84.8 ± 15.7 mm^2^, and in the postfixed group was 137.2 ± 19.5 mm^2^, and there was no significant difference between distribution groups (Figure 8) (*p < *0.001).

## Discussion

This study is the first investigation to combine cadaveric dissections with morphometric radiologic analyses and 3D simulations to examine the superior hypophysial artery with a focus on its aneurysms. Here, we first described relevant anatomy using cadaveric dissections. Then, we presented morphometric measurements appropriate for ipsilateral and contralateral approaches for SHA aneurysms. Finally, we provided virtual 3D simulations of surgical scenarios using morphometric data to inform surgical decision-making.

The superior pituitary artery was first described by von Lusckha (13). Dawson (14) detailed information on the SHA anatomy and reported that it originated from the ICA. Over time, information has revealed that SHA is actually a vascular complex originating from the medial aspect of the segment of the ICA between the ophthalmic artery and the posterior communicating artery (15–17). Recently, the term primary and secondary SHA was used for the first time in the study of Truong et al. (18), in which 110 SHAs originating from 60 ICAs were examined in the endoscopic study in which they examined the surgical anatomy of SHA. According to their findings, two SHAs, proximal and distal, were detected at a rate of 70%. The primary term was used for proximal SHA that feeds the optic nerve, optic chiasm, and infundibulum and travels in the preinfundibular space; the secondary term for SHA that feeds the infundibulum, tuber cinereum, optic tracts, and mammillary bodies and extends in the retroinfundibular or parainfundibular space. Moreover, they stated that there is a single SHA of 18.3% and a third SHA of 8.3%. In addition, they stated that the primary SHA originates from the ophthalmic segment of the ICA at a rate of 1/3 and the clinoidal segment of the ICA at a rate of 2/3 from the distal of the distal dural ring and from the proximal of the distal dural ring, and even half of them originate from the carotid cave. They showed that it has complicated and variational anatomy (18).

The complicated anatomy of SHA is a factor that makes surgery challenging. Complex procedures such as the complexity of the surgical anatomy of the area of SHA, the proximity of SHA to important neurovascular structures in the suprasellar area (optic nerve, chiasm, pituitary stalk, other arterial branches), the need for anterior clinoidectomy to provide proximal control of the ICA and to define the proximal aspect of the aneurysmal neck, optic nerve decompression and mobilization, and opening of the carotid collar are the factors that increase the degree of surgical difficulty in aneurysms (19). In addition, the authors mentioned the problem of accessing the infrachiasmatic space and the difficulty in dissection of SHA (14, 17). In our study, similar to the literature, it was found that it was not possible to provide proximal control in large aneurysms, especially in the contralateral approach.

One of the most important complications that develop due to these difficulties is visual deficits. In two cases presented by El Refaee et al. (20), SHA was occluded during SHA aneurysm surgery, and it was reported that superior quadranopsia developed in one of the patients. Johnson et al. (21) stated that homonymous hemianopsia developed after the operation in a patient who underwent SHA aneurysm embolization. Horiuchi et al. (22) stated that a visual deficit of 13% was observed in a series of 70 patients with SHA aneurysms, and it was commented that unilateral sacrification could not always be performed in SHA aneurysms.

Another problem in SHA aneurysms is the direction of the surgical approach. The common approach in ophthalmic segment aneurysms of the ICA is the ipsilateral pterional approach (23, 24). On the contrary, some authors suggest that surgery should be performed from the contralateral side in paraclinoid and ophthalmic ICA, especially in the ophthalmic segment and SHA aneurysms, since the aneurysm originates from the medial wall of the ICA, but they state that the problem of proximal control also poses a serious disadvantage (10, 11, 25, 26). In the presence of a bilateral aneurysm, a bilateral approach with unilateral craniotomy is recommended (11, 26–28). Finally, another type of surgical approach used in ophthalmic ICA aneurysms is the subfrontal interhemispheric approach (10).

The distance to the lesion and the prechiasmatic space are very important in the contralateral approach. We think that the type of chiasm is one of the most important points in the contralateral approach. In the study of de Oliveira et al. (25) on the contralateral approach to aneurysms, it was stated that the preference for the contralateral approach in ophthalmic ICA aneurysms depends on the size and projection of the aneurysm and its relations with the optic nerve, carotid artery, and anterior clinoid process. It has been stated that anatomical variations of the chiasm, such as the prefix chiasm, may interfere with the contralateral approach since access to the aneurysm is made between the optic nerves. Ophthalmic ICA aneurysms are divided into four types according to their projections, and the most common type is the subchiasmal type that projects medially and originates from the medial surface, but Nishio et al. (10) also drew attention to the effect of the proximity of the neck of the aneurysm to the exit point of the ophthalmic artery on surgery. They stated that the inability to see the origin of the ophthalmic artery in ipsilateral surgery complicates the ipsilateral approach. Since the ipsilateral optic nerve blocks the view of the origin of the ophthalmic artery and the medial aspect of the ipsilateral ICA, mobilization of the optic nerve is necessary to view these areas. For this reason, it has been reported that it is necessary to open the roof of the optic canal, remove the anterior clinoid process and tuberculum sella, and in some cases even a part of the optic nerve. Because of these maneuvers, postoperative visual impairment may be a serious problem in the ipsilateral approach (10). In the contralateral approach, the medial and inferior aspects of the ophthalmic segment of the ICA and the origin of the contralateral ophthalmic artery can be seen without the need for manipulation of the optic nerve; therefore, the aneurysm originating from the anterior, medial, and inferior of the ophthalmic ICA can be reached without causing visual impairment with a contralateral approach (10). Despite these advantages, the contralateral approach may not be suitable for large and ruptured aneurysms. Another handicap of the contralateral approach is that it can cause bilateral olfactory nerve damage and develop a permanent sense of smell (10). In the setting of large and anteromedially protruding aneurysms and prefixed chiasm, which prevents a contralateral approach, a combined contralateral pterional and interhemispheric approach has also been proposed (29). However, if the aneurysm neck is small and there is a space between the anterior wall of the aneurysm and tuberculum sellae, it might be possible to clip even giant aneurysms through a contralateral approach without optic injury (30).

Carotid cave aneurysms, one of the segments where SHA can originate, can be given as an example of a special type of intracranial aneurysm where the surgical approach is the most challenging. In the carotid cave aneurysm series of 31 cases by Sheikh et al. (28), the contralateral approach was applied to four patients, and the contralateral approach was recommended in this type of aneurysm because it does not cause optic damage and can be easily applied in multiple and bilateral aneurysms. Kakizawa et al. (7) reported the necessary parameters for the contralateral approach for ICA-ophthalmic aneurysms. They identified that the direction of the aneurysm from ICA on the anteroposterior angiogram and the distance between the medial aspect of the distal dural ring and the proximal aneurysm neck on the lateral angiogram were two important factors in predicting the difficulty in the contralateral approach.

Although there are many studies on ophthalmic ICA aneurysms in the literature, there are limited studies on isolated SHA aneurysms. Although it has been stated that most paraclinoid aneurysms are associated with SHA, occlusion may not cause any deficit during the treatment of SHA aneurysm, and therefore, SHA can occasionally be sacrificed in some cases; there is no way to predict whether or not an obliteration of SHA would cause visual deficits (31). Despite the peculiarities of SHA aneurysms and their differences from other ophthalmic segment aneurysms, there is limited literature on isolated SHA aneurysms. Neurosurgeons dealing with these aneurysms need more focused guidance for these pathologies, considering the complex anatomy of SHA and the expectation of no deficits in the postoperative period in unruptured aneurysms. Most of the available studies on isolated SHA aneurysms are clinical series presentations. In the series of eight cases by Chen et al. (32), surgery was performed with the contralateral approach in nonruptured SHA aneurysms, and it was stated that no complications, including the optic deficit, developed and the contralateral approach was safe and technically possible in unruptured aneurysms. Godbole et al. (19) presented a new classification based on parameters such as aneurysm size, origin, relationship with important structures, and type (saccular, fusiform) over 14 disease series including ruptured and unruptured SHA aneurysms. They argued that detailed information about anatomical variations is necessary in order to achieve satisfactory results in their study, where they stated that, according to their results, there was no interpretation of the optic deficit in five patients, optic deficit was absent in seven patients, and optic deficit occurred in two patients (19). In parallel to Godbole et al. (19), we identified conditions and limitations of ipsilateral and contralateral approaches to SHA aneurysms combining anatomical study with morphometric analyses and 3D simulations. The findings of the study may aid neurosurgeons in choosing the proper approach for SHA aneurysms. Hereby, we stress the utmost importance of patient-specific radiological anatomy. Indeed, even the intraoperative monitoring of visual evoked potentials might not be sufficient to prevent visual deficits in these surgeries (33–36).

### Strengths and Limitations

This study combined different methodologies to study the microsurgical anatomy of SHA and its aneurysms. Robust morphometric analysis and its integration with the 3D virtual model simulation are its strengths. However, it has certain limitations, too. First, we did not study anatomic variations of SHA. Second, we did not examine angiographic variations related to SHA aneurysms. Third, although simulations can be potentially enriched by introducing endless variations, they only provide anatomic relationships but do not inform about the challenges faced in real-world settings such as adherence of aneurysm wall to adjacent structures and the extent to which optic nerves can be mobilized. Further studies with the incorporation of angiographic images into simulations can better inform decision-making processes.

## Conclusion

This study is the first investigation to combine cadaveric dissections with morphometric radiologic analyses and 3D simulations to examine superior hypophysial artery with a focus on its aneurysms. Anatomic dissections along with 3D virtual model simulation and illustrations demonstrated that (i) the contralateral approach would potentially allow for proximal control and neck control/clipping in smaller SHA aneurysm with relatively minimal retraction of the contralateral optic nerve in the setting of pre- or normofixed chiasm and (ii) the ipsilateral approach requires anterior clinodectomy and optic unroofing with considerable optic nerve mobilization to effectively control proximal ICA and clip the aneurysm neck. Careful examination of preoperative imaging studies and patient-specific 3D modeling may further help surgeons choose the right approach to tackle SHA aneurysms.

## Data Availability

The raw data supporting the conclusions of this article will be made available by the authors without undue reservation.
